# Clinical value of noninvasive lens advanced glycation end product detection in early screening and severity evaluation of patients with diabetic kidney disease

**DOI:** 10.1186/s12882-023-03428-3

**Published:** 2023-12-19

**Authors:** Xiaodi Zheng, Yuan Gao, Yuhong Huang, Ruihua Dong, Mengxue Yang, Xuemeng Zhang, Miao Zeng, Rui Zhang, Yueyue Wu, Zhiyan Yu, Jun Liu, Bingbing Zha

**Affiliations:** 1 Department of Endocrinology, Shanghai Fifth People’s Hospital, Fudan University, Shanghai, China; 2 Center of Community-Based Health Research, Fudan University, Shanghai, China; 3 General Practice Clinic, Pujiang Community Health Service Center in Minhang District, Shanghai, China; 4 Key Lab of Public Health Safety of the Ministry of Education, School of Public Health, Institute of Nutrition, Fudan university, Shanghai, China; 5 Department of Infectious Diseases, Shanghai Fifth People’s Hospital, Fudan University, Shanghai, China

**Keywords:** Advanced glycation end products, Type 2 diabetes, Ratio of microalbuminuria to creatinine, Glomerular filtration rate

## Abstract

**Background:**

Advanced glycation end products (AGEs) deposited in the lens are correlated with those in the kidneys, indicating a possible value in evaluating diabetic kidney disease (DKD). This study explored the value of noninvasively measuring lens AGEs to diagnose and evaluate the severity of diabetic nephropathy in patients with type 2 diabetes mellitus (T2DM).

**Methodology:**

A total of 134 T2DM patients admitted to the Fifth People's Hospital of Shanghai from March 2020 to May 2021 were selected randomly. Patients were divided into low-, medium-and high-risk groups according to the risk assessment criteria for DKD progression and into DKD and non-DKD (non-DKD) groups according to the Guidelines for the Prevention and Treatment of Diabetic Nephropathy in China. The concentrations of noninvasive AGEs in the lens in all the groups were retrospectively analyzed.

**Results:**

The concentration of noninvasive lens AGEs in the high-risk patients, according to the 2012 guidelines of the Global Organization for Improving the Prognosis of Kidney Diseases, was significantly higher than that in the remaining groups. Regression analysis suggested the value of lens AGEs in diagnosing DKD and evaluating DKD severity. Cox regression analysis indicated that the noninvasive lens AGE concentration was positive correlated with the course of disease.

**Conclusion:**

The receiver operating characteristic (ROC) curve suggested that using noninvasive lens AGE measurements has clinical value in the diagnosis of DKD (area under the curve 62.4%,95% confidence interval (CI) 52.4%–73.9%, *p =* 0.014) and in assessing the severity of DKD (area under the curve 83.2%, 95% CI 74.1%–92.3%, *P <* 0.001). Noninvasive lens AGE testing helps screen T2DM patients for DKD and evaluate the severity of DKD.

## Introduction

Diabetic kidney disease (DKD) is one of the complications of diabetes and an important cause of end-stage renal disease. Approximately 31.3% of new end-stage renal disease cases in 2015 originated from DKD [1]. The incidence of chronic kidney diseases related to diabetes has increased steadily in recent years [2]. In China, over 141 million adults have diabetes mellitus (DM) [3], and in 2019, there were 4,613 cases of chronic kidney disease (CKD) related to DM per 100,000 people [4]. With the prolongation of the disease, the total number of CKD patients will continue to increase. Thus, it is particularly important to identify high-risk patients with DKD and intervene as soon as possible.

China’s Guidelines for the Prevention and Treatment of Diabetic Nephropathy (2021 edition) [5] propose that the ratio of urinary albumin to creatinine (UACR) or 24-h urinary albumin excretion rate (UAER), estimated glomerular filtration rate (eGFR) and renal biopsy can be used for the diagnosis of DKD. However, to obtain the above data, invasive operations are needed, and kidney biopsy is especially invasive. Kidney biopsy can lead to kidney hematoma (11%) and gross hematuria (3.5%), and in severe cases, blood transfusion (1.6%) or even hemostasis (0.3%) [6]. Therefore, safer, more convenient and noninvasive indicators for early screening of DKD and risk classification are needed to help clinicians formulate personalized treatment plans and reasonably allocate limited medical resources. The exploration of early screening methods and models for DKD continues. In this field, eGFR and UACR are early laboratory markers that can be used for early screening of DKD [7]. However, clinically, DKD is susceptible to interference by human immunodeficiency virus infection, chronic liver diseases, cardiovascular diseases and other diseases, and it is difficult to measure it accurately [8]. In recent years, some scholars have found that laboratory markers, such as exosomes, can be used for early screening of DKD [9]. Renal function can also be evaluated by medical instruments, such as renal ultrasound and renal CT [10, 11]. However, these methods are only worthwhile after changes in kidney structure or function. Therefore, these are rarely used for the early screening of DKD clinically. Other studies have suggested that incorporating age, sex, smoking history, systolic blood pressure, and course of diabetes into the DKD screening model has good clinical application value [12]. The shortcoming of these indices, such as systolic blood pressure, is that they differ between day and night, and they are also easily perturbed by one's own emotions, medication and other external environmental factors [13].

Advanced glycosylation end products (AGEs) were reported by French chemist Maillard early in the last century. They are a general term for stable end products formed by a series of reactions between the free amino groups of proteins, amino acids and other substances and the carbonyl group of reducing sugars. They are autofluorescent, highly stable, and not easily decomposed in the human body. AGEs are deposited in the kidney [14], the lens of the eye [15, 16] and other tissues and organs, where they damage their normal physiological functions. AGEs make up for the above disadvantages with their stability. AGEs have been considered one of the factors leading to renal fibrosis. However, they can also induce the production of a variety of cell growth factors and oxidative stress and promote the release of reactive oxygen species and inflammatory mediators [17, 18]. Scholars have adopted noninvasive skin AGE detection technology for diabetes screening. However, skin AGE detection technology is easily affected by skin care products, and this effect is not easily prevented by washing hands or wiping with ethanol [19]. In an experiment targeting 100 subjects, Wang et al. tested the normal population and the population with diabetes using lens AGE detection technology and showed a significant difference between the fluorescence deviations of the populations with and without diabetes. This finding supports the feasibility of lens autofluorescence screening in subjects with undiagnosed diabetes [20]. In our previous studies, animal experiments have shown [21] that the deposition of AGEs in the kidney and their deposition in the lens were positively correlated. Therefore, we intended to indirectly measure the levels of AGEs deposited in the kidney by measuring AGEs in the lens by a noninvasive detection method in the search for a noninvasive screening technology or index for DKD. Many scholars have measured AGEs before, but the use of noninvasive testing of AGEs to assess the severity of DKD has not been reported [22]. According to Prasad K’s study, AGEs are involved in the pathogenesis of CKD and may serve as risk factors for CKD [23].

The purpose of this study was to explore the clinical application value of noninvasive lens AGE measurement to screen for DKD and assess the severity of DKD. The findings of this study will help to explore the pathogenic mechanisms of CKD.

## Data and methods

### General information

A total of 212 patients who were definitively diagnosed with DM in the Fifth People's Hospital of Shanghai Affiliated to Fudan University from March 2020 to May 2021 were screened randomly. All DM patients were diagnosed and treated according to guidelines [5].

**Fig. 1:**
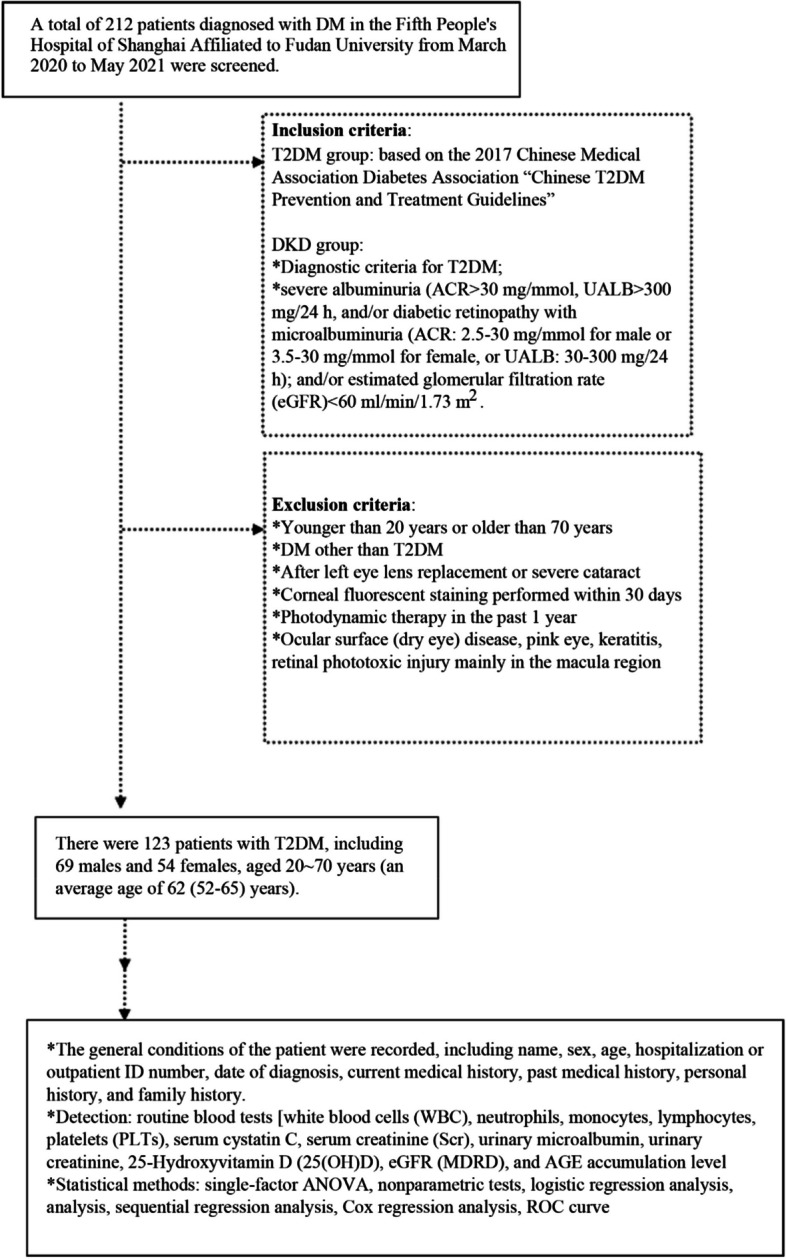
Experimental flow chart

Exclusion criteria: 1) less than 20 years old or more than 70 years old; 2) type 1 diabetes, gestational diabetes, or special diabetes; e) previous intraocular lens transplantation in the left eye or cataract of Grade 3 or more; 4) corneal fluorescence staining within the last 30 days; 5) fluorography within the last six months; 6) photodynamic therapy in the past year; 7) ocular surface (dry eye) diseases, pinkeye, keratitis, or retinal phototoxicity injury mainly caused by macula, etc. A total of 123 patients with type 2 DM (T2DM) were enrolled in the study, including 69 males and 54 females, with an average age of 62 (52–65) years, ranging from 20 to 70 years. The flow diagram of this study is shown in Fig. 1.

All procedures involving human participants in this study were performed in accordance with the 1964 Declaration of Helsinki and its subsequent amendments and approved by the Ethics Committee of the Fifth People's Hospital of Shanghai Affiliated to Fudan University (approval no. 2021–142). Written informed consent was obtained from each participant.

### Data collection

#### Medical history collection

A unified epidemiological questionnaire was adopted and investigated. The following general conditions of the patient were recorded: name, sex, age, hospitalization or outpatient ID number, date of diagnosis, current medical history, past medical history, personal history, and family history.

#### Measurement of anthropometric parameters

Height, weight and blood pressure were measured, and body mass index (BMI) was calculated as weight/height squared (kg/m^2^). When being weighed, the patient was wearing only underwear. The weighing scale was corrected to 0.1 kg, and the height measurement was accurate to 0.1 cm. Prior to blood pressure measurement, patientswere not allowed to smoke or drink coffee within half an hour and they were asked to rest in a chair with a backrest for at least 15 min in a quiet environment. For each patient, measurement was performed on the right upper arm thrice,with the patient in a supine or sitting position, and an average was obtained.

#### Detection of biochemical indices

A Sysmex XN-9000 Automatic Hematology Analyzer was used to run routine blood tests [white blood cells (WBCs), neutrophils, monocytes, lymphocytes and platelets (PLTs)]. Serum cystatin C, serum creatinine (Scr), urinary microalbumin, C-peptide 2 h after meal, fasting C-peptide and urinary creatinine levels were measured.25-Hydroxyvitamin D(25(OH)D) was detected by a Roche Cobase 601 fully automatic electrochemiluminescence immunoassay analyzer.

#### *eGFR calculation * [24]

eGFR(modification of diet in renal disease) = 186 × (Scr)^−1.154^ × (age)^−0.203^ × 0.742 (if female) × 1.233 (if Chinese).

#### AGEscan fluorescence detection

AGE scan fluorescence detection was performed by qualified medical staff after standardized training. A noninvasive lens AGE scan fluorescence detector (model: AGE scan) (provided by Sannuo Biosensor Co., Ltd. Changsha, Hunan province, China) was used. The AGEscan emits a beam of blue light, which is safe for human body, to irradiate the lens of the left eye. AGEs in the lens are thus excited to produce fluorescence, which can then be detected by the instrument. Finally, a specific value will be assigned according to the detected intensity of fluorescence, which is positively correlated with the level of AGE accumulation.

### Grouping

#### DKD progression

According to the 2012 guidelines of the Global Organization for Improving the Prognosis of Kidney Diseases (KDIGO) [25] and the Guidelines for the Prevention and Treatment of Diabetic Nephropathy in China (2021 edition) [5], patients with DKD were stratified according to their CKD progression risk and their frequency of visits for GFR and UACR. According to the “Prognosis of CKD by GFR and Albuminuria Categories: KDIGO 2012” [25], all patients were divided into four groups (green, yellow, orange and red). The green group was a low-risk group (Group 1), the yellow group was a medium-risk group (Group 2) and the orange group was a high-risk group (Group 3). In this study, no patient meeting the criteria of extremely high risk was enrolled, so the red group was absent.

#### DKD

All patients were divided into a diabetic nephropathy group (DKD, UACR ≥ 30 mg/g and/or EGFR < 60 ml/min * 1.73 m2) and a non–diabetic nephropathy group (non-DKD, UACR < 30 mg/g and EGFR ≥ 60 ml) according to the Guidelines for the Prevention and Treatment of Diabetic Nephropathy in China (2021 edition).

### Statistical methods

SPSS 25.0 software was used for analysis, and the Kolmogorov–Smirnov (K-S) test was used to analyze whether the data satisfied a normal distribution. Data are expressed as the mean standard deviation ($\overline{x }$±s) when the normal distribution was met and as median (interquartile spread) [M(P25, P75)] when the normal distribution was not met. Single-factor ANOVA, nonparametric tests and analysis of variance were used to evaluate the differences between groups after they were grouped by each index. Logistic regression analysis and Cox regression analysis were performed on the data tested by parallel lines. The receiver operating characteristic (ROC) curve was drawn for prognostic analysis. *P <* 0.05 was considered statistically significant.

## Results

According to the classification criteria of the progressive risk assessment of DKD recommended by the KDIGO 2012 guidelines [25] and China’s Guidelines for the Prevention and Treatment of Diabetic Nephropathy (2021 edition) [5], 76 patients were low-risk (Group 1), 35 patients were medium-risk (Group 2) and 12 patients were high-risk (Group 3). The three groups differed significantly in the values of AGEs, cystatin C, 25-hydroxyvitamin D, fasting C peptide, 2-h postprandial C peptide, leukocytes, neutrophils, monocytes, and the neutrophil-to-lymphocyte ratio (NLR) (*P <* 0.05, see Table 1 for details).

**Table 1:** DKD progression risk assessment in the 3-group stratification

	Group 1	Group 2	Group 3	p
Age (years)	59(52–66)	59(45–66)	63(53–65)	0.755
Course of disease (years)	8(4–15)	10(4–16)	11(7–16)	0.311
Body mass index (kg/m^2)	24.4(22.5–27.7)	25.5(21.2–27.0)	23.4(21.6–27.2)	0.794
AGEs detection value of noninvasive lens	0.283 ± 0.069	0.290 ± 0.095	0.376 ± 0.057^ab^	0.001
Cystatin c (mg/L)	0.90(0.78–0.99)	0.80(0.64–0.97)	1.11(0.96–1.27)^ab^	0.014
25-hydroxyvitamin-D (nmol/L)	48.2(35.9–60.3)	42.7(36.3–66.0)	28.7(24.6–35.9)^ab^	0.001
Glycosylated hemoglobin (%)	8.4(7.0–9.6)	10.0(8.9–12.1)^a^	9.6(9.1–11.0)	0.001
Fasting C-peptide (nmol/L)	0.65(0.47–0.80)	0.51(0.30–0.80)	0.47(0.24–0.98)	0.116
C-peptide 2 h after meal (nmol/L)	1.78(1.13–2.31)	0.75(0.47–1.79)^a^	0.75(0.45–1.75)^a^	< 0.001
Fasting blood glucose (mmol/L)	7.0(5.6–8.9)	7.2(6.0–11.1)	8.1(5.1–10.2)	0.129
2 h postprandial blood glucose (mmol/L)	11.9(9.3–15.4)	13.0(8.6–17.4)	7.7(9.1–15.1)	0.083
Fasting insulin (pmol/ml)	47.6(29.2–68.8)	41.7(16.6–68.80)	66.0(26.4–101.2)	0.388
Insulin 2 h after meal (pmol/ml)	261.5(138.8–383.6)	147.4(79.6–276.8)^a^	167.0(66.8–360.1)	0.017
Hemoglobin (g/L)	135.9 ± 13.2	133.6 ± 13.9	134.0 ± 24.6	0.340
Blood platelets (*10^9/L)	212.3 ± 56.8	225.7 ± 63.7	241.9 ± 71.8	0.398
NLR	1.87(1.25–2.28)	2.29(1.38–3.46)^a^	2.33(1.72–2.89)	0.042
PLR	120(89–155)	126(99–169)	126(101–161)	0.444
NMR	6.74(5.52–8.84)	8.29(6.78–9.83)^a^	7.27(6.30–8.78)	0.097
White blood cells (*10^9/L)	5.66(4.84–6.59)	5.78(5.33–6.71)	6.72(5.97–7.73)^a^	0.041
Neutrophils (*10^9/L)	3.07(2.43–3.96)	3.71(3.01–5.06)^a^	4.21(3.52–5.10)^a^	0.004
Monocytes (*10^9/L)	0.44(0.35–0.60)	0.42(0.37–0.54)	0.56(0.43–0.62)	0.155
Lymphocytes (*10^9/L)	1.82(1.54–2.19)	1.76(1.28–2.42)	1.88(1.42–2.24)	0.591
Red blood cells (*10^12/L)	4.48 ± 0.41	4.41 ± 0.48	4.51 ± 0.90	0.505

Note: Group 1: low risk group; Group 2: medium risk group; Group 3: high risk group

*NLR* neutrophil to lymphocyte ratio, *PLR* platelet to lymphocyte ratio, *NMR* granulocyte to monocyte ratio

^a^*P <* 0.05 vs. group 1

^b^*P <* 0.05 vs. group 2

**Fig. 2:**
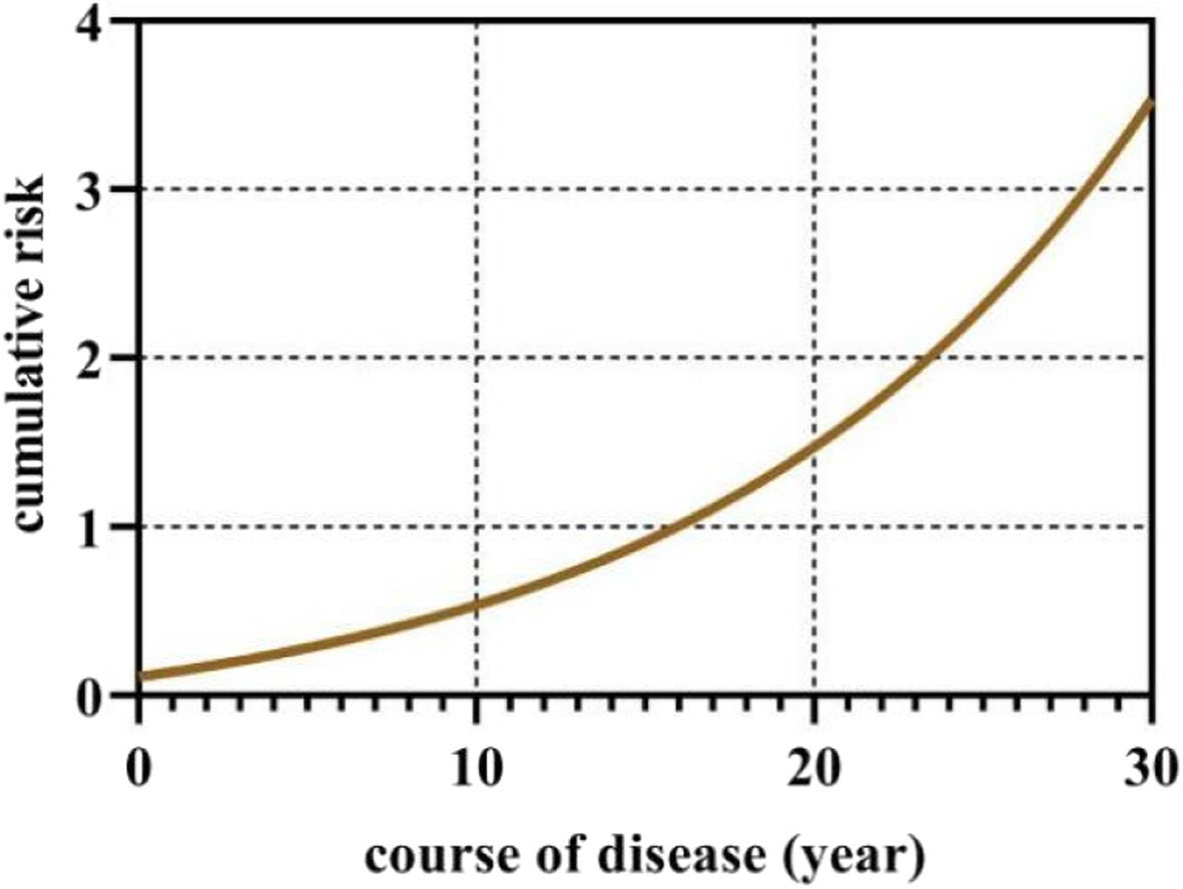
Correlation between the course of disease and DKD risk level

As the noninvasive lens AGE concentration went from low to high, the risk assessment of DKD progression also changed from low risk to high risk, which suggests that lens AGE concentration is related to the severity of DKD (Fig. 2). We speculated that noninvasive lens AGEs could be used to evaluate the severity of DKD. Linear regression analysis suggested that the values of noninvasive lens AGEs and cystatin C were statistically significant predictors of the severity of DKD (*P <* 0.05, *R*^2^ = 0.996) (Table 2).

**Table 2:** Value of different indicators in evaluating the severity of DKD

Indicator	Coefficient of regression	*P* value	95% CI	
			Lower limit	Upper limit
Noninvasive lens AGE level	2.333	0.002	0.895	3.772
Cystatin C (mg/L)	0.640	0.018	0.112	1.168

When we divided all T2DM patients into the DKD group (ACR ≥ 30 mg/g and/or EGFR < 60 ml/min * 1.73 m^2^) and the non-DKD group (ACR < 30 mg/g and EGFR ≥ 60 ml/min * 1.73 m^2^) for binary analysis, the results showed that the concentration of AGEs in the lens was statistically significant (*P <* 0.05) in distinguishing the DKD group and the non-DKD group (Table 3).

**Table 3:** Value of different indices in the diagnosis of DKD

Group	Noninvasive lens AGE level	Cystatin C (mg/L)
Non-DKD group	0.283 ± 0.069	0.89(0.79–0.99)
DKD group	0.313 ± 0.094	0.96(0.74–1.12)
*P* value	0.014	0.115

Note: *DKD group* diabetic nephropathy group, *Non-DKD* non–diabetic kidney disease group

Cox regression analysis suggested that there was a significant relationship between the risk level predicted by AGEs in the lens and the course of disease (*P <* 0.05) (see Table 4 for details).

**Table 4:** Correlation between the course of disease and different renal function indices

Group	*P* <	95% CI	
		Lower limit	Upper limit
Noninvasive lens AGE level	0.014	0.001	0.455
Cystatin C (mg/L)	0.068	0.131	1.077

**Fig. 3:**
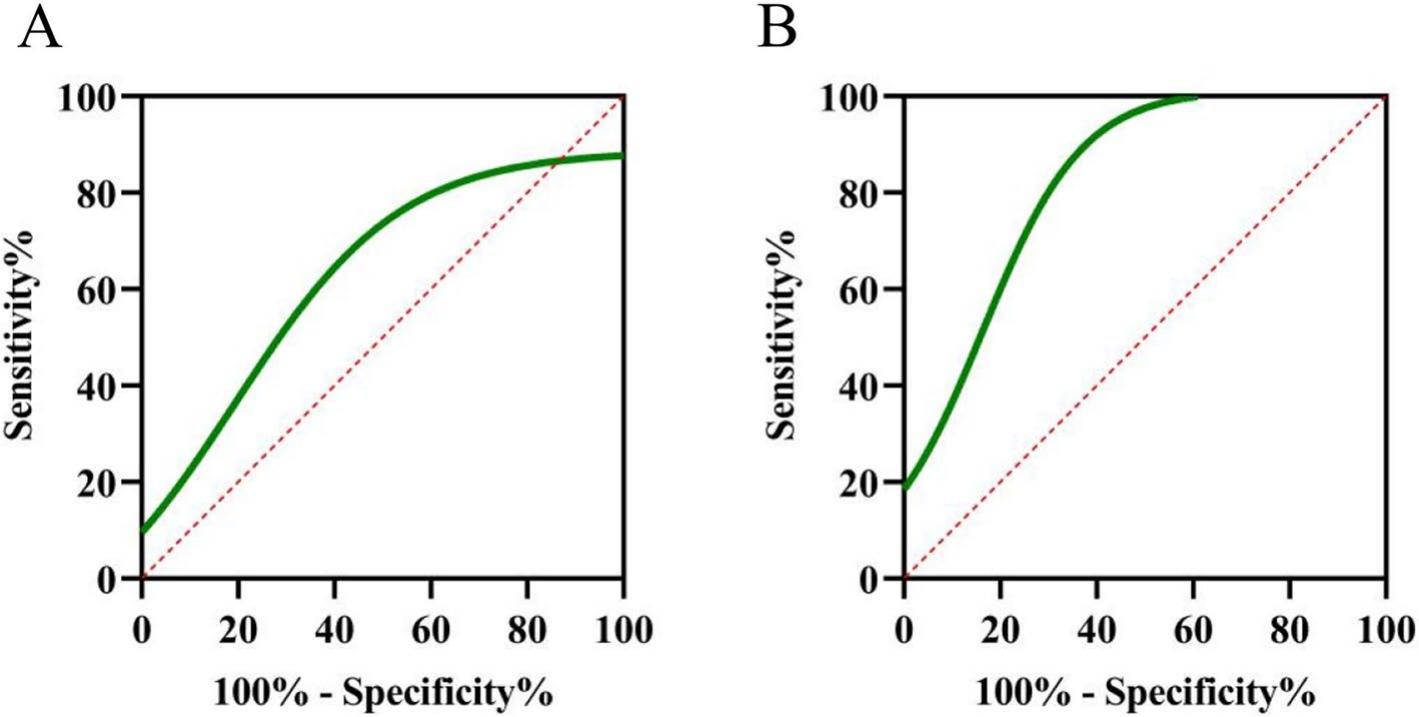
Value of different grouping methods in noninvasively detecting AGEs in the lens. A: Value of noninvasive lens AGE measurement in the diagnosis of DKD;B: Value of noninvasive lens AGE measurement in classifying DKD severity

The statistical results shown in Table 1 indicate that there was no significant difference between Group 1 and Group 2; therefore, Group 1 was combined with Group 2. The ROC curve was used to evaluate the value of noninvasive lens AGE measurement in the diagnosis of DKD in patients with T2DM (Fig. 3A). The area under the curve was 62.4%, *p =* 0.014, and the 95% CI was 52.4%–73.9%. The ROC curve was used to evaluate the severity of DKD in T2DM patients through noninvasive lens AGE measurement (Fig. 3B). The area under the curve was 83.2% (95% CI 74.1%–92.3%, *P <* 0.001).

## Discussion

There are few reports on the use of AGEs for the screening and assessment of the severity of DKD, and there are no reports regarding the use of noninvasive screening methods for the early screening and severity rating of DKD in T2DM patients. Therefore, the purpose of this study was to investigate the clinical value of a lens AGE noninvasive measurement in screening for DKD and assessing the severity of DKD and to provide a new theoretical basis for the clinical screening of DKD.

In the present cross-sectional study, we found that according to the risk assessment of DKD progression recommended by the KDIGO 2012 guidelines, the concentration of noninvasive lens AGEs in T2DM patients classified in the high-risk group was significantly higher than that in the low-risk and medium-risk patients. The results show that noninvasive lens AGE measurements can be used to aid in the diagnosis of DKD. We also found that the concentration of noninvasive lens AGEs was an independent risk factor for the severity of DKD and had a high regression coefficient, which indicates that it has certain clinical value in evaluating the severity of DKD. Rezaei Mohaddeseh et al. [26] found that plasma AGE concentrations were positively correlated with diabetic retinopathy, but no correlation was found between plasma AGE concentrations and DKD. In this study, by measuring the contents of AGEs in the lens, we found that they were correlated with DKD. There seems to be a contradiction between the results of the two studies, but the sites of AGEs collected by the two studies are different. We speculate that the deposition of AGEs in different parts of the body is not balanced, and this difference will lead to differences in the onset order of different complications of DM. More studies are needed to verify this hypothesis. It is undeniable that the accumulation of AGEs in the body is positively correlated with the occurrence of complications of DM [27]. Thus, the change in AGE levels can be used to determine the stage of the disease and evaluate the curative effect [28].

Our research shows that with the prolongation of the course of disease, the risk of DKD diagnosis in T2DM patients will increase, but the content of cystatin C in the human body does not increase with the prolongation of the disease course. Previous research has shown that the deposition of AGEs in vivo is positively correlated with age and the course of disease in patients [29], which is similar to the results of our study. Using noninvasive lens AGEs is more helpful for the classification of DKD severity than detecting and diagnosing whether T2DM patients have DKD. Our previous study [20] initially confirmed the value of noninvasive lens AGE detection in the diagnosis of DKD. We conducted this study to further explore whether it has any other value for patients with T2DM, and it was again verified that lens AGE noninvasive detection technology can be used to screen DKD. The research results show that according to the noninvasive lens AGE detection value concentration from low to high, the risk assessment of DKD progression also changes from the low-risk group to the high-risk group. Noninvasive lens AGE concentration can be used to assess the severity of DKD.

We also found that the number of white blood cells, neutrophils and monocytes were significantly higher in the high-risk patients with DKD than in the low-risk patients (*P <* 0.05). PLR and NLR tended to increase with the risk level, but this difference was not significant (*P >* 0.05). This result is consistent with that reported by Prasad K et al. [11]. We speculated that this might be related to the fact that the high level of AGEs in the body directly or indirectly promotes the chronic systemic inflammatory response. Many clinical studies have shown that inflammatory indicators, such as the PLR and NLR, are widely used to assess the mortality of peritoneal dialysis patients, the prognosis of tumor patients, the severity and rate of acute coronary syndrome and other aspects [30–32]. This suggests that the above inflammation-related indicators might also be used as components of the DKD prediction model. Testing such a hypothesis requires in-depth study.

This study has some limitations. Due to the small sample size, the number of cases was not well balanced between groups for statistical analysis. This study lacks relevant data onpatients with an extremely high risk of DKD. It was also only a single-center study. In the future, we will conduct a multicenter study, expand the sample size and explore in depth the common rules of test data to lay a research foundation for finding a prediction model that accurately screens for DKD in T2DM patients.

This study preliminarily confirmed that noninvasive lens AGE detection technology may serve as a rapid and highly acceptable method to evaluate whether T2DM patients have DKD and the severity of their DKD. The findings of this study will help clinicians to better identify, diagnose and treat DKD, to better guide patients’ treatment, and to more rationally allocate medical resources.

## Data Availability

The datasets generated during and/or analysed during the current study are available from the corresponding author on reasonable request.
